# Non-surgical spinal cord infarction: case series & long-term follow-up of functional outcome

**DOI:** 10.1038/s41394-024-00665-y

**Published:** 2024-10-24

**Authors:** Fionán McBride, Jane Anketell, Gavin V. McDonnell, Suzanne Maguire, Karen M. Doherty

**Affiliations:** 1 Queen’s University Belfast, Belfast, N.Ireland UK; 2 Spinal Cord Injuries Unit, Musgrave Park Hospital, Belfast, N.Ireland UK; 3 Department of Neurosciences, Royal Victoria Hospital, Belfast, N.Ireland UK

**Keywords:** Diagnosis, Neurological manifestations, Rehabilitation, Spinal cord diseases, Stroke

## Abstract

**Introduction:**

Spinal cord infarction is a rare but often devastating disorder. The pathogenesis of most non-surgical cases involves atherothrombosis and treatment with anticoagulation and antiplatelet agents may be indicated. Functional recovery in most cases is poor. We describe five cases of spinal cord infarction and provide details on their functional outcomes after long-term (>10 years) follow-up.

**Case presentation:**

A 28-year-old female presented at 16 weeks gestation with chest and back pain and paraesthesia in her fingers. Magnetic resonance imaging on admission revealed a spinal cord lesion extending from C5-T8. She was treated with anticoagulation and rehabilitation. Six years following presentation she was able to return to work. A 42-year-old male experiencing central chest pain and leg weakness was initially diagnosed as having acute coronary syndrome. Following discharge, he was re-admitted with urinary retention and leg weakness. Magnetic resonance imaging revealed a spinal cord lesion extending from T4 to T7. He was treated with anticoagulation, and eight months following presentation he regained full muscle strength but required intermittent self-catherisation. Three further cases are described.

**Discussion:**

The aetiology of non-surgical spinal cord infarction is not always evident, but is commonly associated with atherothrombosis. There are often delays in making a diagnosis, but early recognition and prompt treatment of spinal cord infarction is essential. Long-term functional outcomes are often poor and typically reflect the severity of initial presentation. This case series is unique as it has one of the longest follow-up periods described in the literature.

## Introduction

The literature invariably reports spinal cord infarction (SCI) as ‘rare’ and considerably lower than that of infarction of the brain [1]. Early studies proposed that anterior spinal artery (ASA) infarction was caused by local disease of the artery secondary to syphilis, vasculitis or atherosclerosis [2]. It is now recognised that infarction in the ASA distribution is more often caused by arterial disease, most commonly the aorta in the setting of arteriosclerosis, dissection or repair of a thoracic or abdominal aneurysm [3–6].

Thrombophilic disorders may have a causative role in spinal strokes affecting patients in younger age groups, especially in those concurrently taking oral contraceptives [7, 8]. Some case series have also found an association between degenerative spinal disease and cord infarction [9].

Historically there have been relatively few studies examining long-term outcome following SCI. Although recent years have seen a growing interest in this area [5, 6, 10–12], the mean follow-up period for functional outcomes in the literature rarely exceeds three years. We report five non-surgical cases of SCI who presented over an eight month period to the Regional Neurology Unit in Belfast with a mean follow-up of 6.5 years. The degree of impairment at presentation and functional outcomes after long-term follow-up for all five cases are summarised in Tables 1, 2. Initial presentations, early clinical and the last documented outpatient review in the Spinal Cord Injuries Unit (SCIU) ten years later has been examined to elicit long-term functional outcomes.

**Table 1:** Comparison of neurological impairment at presentation and disability at long-term follow-up.

Case	Age at presentation	Sex	ASIA impairment on presentation	mRS score at last follow-up
1	28	F	C	2
2	42	M	D	1
3	64	M	A	5
4	53	F	C	3
5	56	M	A	4

ASIA Grade: A = Complete impairment, no motor or sensory function below the lesion; C = Incomplete impairment, motor function preserved but more than half of the key muscles below the lesion have a muscle grade less than 3; D = Incomplete impairment, motor function preserved and at least half of the key muscles below the lesion have a muscle grade of 3 or more.

mRS Score: 5 = Severe disability, bedridden, incontinent, and requiring constant nursing care and attention; 4 = Moderately severe disability, unable to walk without assistance, and unable to attend to own bodily needs without assistance; 3 = Moderate disability, requiring some help, but able to walk without assistance; 2 = Slight disability, unable to carry out all previous activities but able to look after own affairs without assistance; 1 = No significant disability despite symptoms, able to carry out all usual duties and activities.

*ASIA* American Spinal Injury Association, *mRS* modified Rankin scale.

**Table 2:** Functional outcomes at long-term follow-up by initial AIS grade.

	All (*N* = 5)	AIS grade		
		A (*n* = 2)	C (*n* = 2)	D (*n* = 1)
Mean follow-up, years (SD)	6.5 (2.5)	5.5 (2.1)	8.3 (2.4)	5.0 (0.0)
Ambulation status % (n)				
Wheelchair-bound	40 (2)	100 (2)	0	0
With assistance	20 (1)	0	50 (1)	0
Independently mobile	40 (2)	0	50 (1)	100 (1)
Catheterisation % (n)				
Indwelling	40 (2)	100 (2)	0	0
Intermittent self-catheterisation	20 (1)	0	0	100 (1)
No catherisation required	40 (2)	0	100 (2)	0
Mortality % (n)	40 (2)	100 (2)	0	0

AIS Grade: A = Complete impairment, no motor or sensory function below the lesion; C = Incomplete impairment, motor function preserved but more than half of the key muscles below the lesion have a muscle grade less than 3; D = Incomplete impairment, motor function preserved and at least half of the key muscles below the lesion have a muscle grade of 3 or more.

*AIS* American Spinal Injury Association (ASIA) Impairment Scale.

## Case presentation

### Case 1

A 28-year-old pregnant female presented to her GP at 16 weeks gestation, with sudden onset chest and back pain and tingling in her fingers. She was briefly assessed before being sent home with a diagnosis of possible carpal tunnel syndrome and mechanical back pain. Upon arriving home she was unable to open the car door with her hands and had difficulty walking to her house. She re-presented to her local hospital and the following day a consultation with the regional Neurology department revealed profound weakness of her hands with grade 0 power in finger flexion and abduction. She had an asymmetrical flaccid paraparesis (right leg Medical Research Council (MRC) grade 0, left leg MRC grade 2), lower limb areflexia and a sensory level to pin prick at C7. There was preservation of proprioception and vibration sensation. With a single neurological level of C7 and fewer than half the muscles below this point grade 3 or more, this injury was classified as American Spinal Injury Association (ASIA) Impairment Scale (AIS) C [13].

**Fig. 1:**
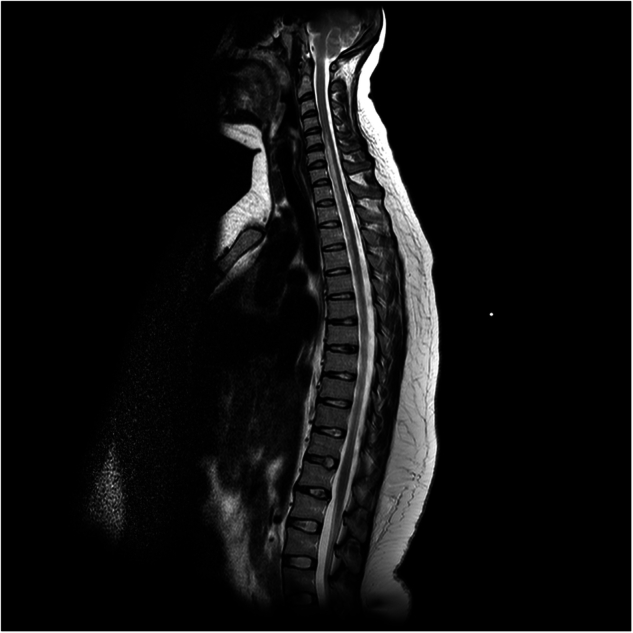
Sagittal T2-weighted image showing increased signal within spinal cord from C5-T8.

A T2-weighted MRI of her spine showed a lesion of abnormal high signal intensity centred anteriorly within the cord extending from C5 to T8—corresponding to the distribution of the ASA (Fig. 1). Magnetic resonance angiography of the aorta revealed that all the major vessels filled normally, with no evidence of a dissection flap. Cerebrospinal fluid analysis and echocardiography were unremarkable. Blood tests, including thrombophilia and vasculitis screens, were normal. She was diagnosed with SCI treated with low molecular weight heparin (LMWH). Her rehabilitation was limited by autonomic dysfunction, which caused significant postural hypotension and required her to be nursed flat for several weeks. She progressed to a normal vaginal delivery of a healthy baby, and when discharged from the SCIU she was able to walk with a walker but her hand function remained poor. She also required an indwelling urinary catheter at that time.

Six years and eight months following presentation her overall function had improved. She could ambulate 200 metres independently. Power had improved in her left hand and she had undergone an opponens plasty of her left hand with success, allowing her useful opposition movement. Her right hand remained dysfunctional, with no finger flexion and evidence of muscle wasting. She continued to have mild neurogenic bladder requiring anticholinergics. However, she no longer required intermittent catheterisation. She managed her bowels on sensation, with intermittent use of laxatives. Upon last review she had returned to work and was continuing to work.

### Case 2

A 42-year-old male developed severe central chest pain and reported having ‘wobbly’ legs while playing football. He admitted to having intermittent chest discomfort and episodes of restless leg sensation in the preceding months, and occasional back pain in the preceding years. He presented to Accident and Emergency (A&E) and was triaged as a possible acute coronary syndrome. After a normal electrocardiogram and a negative 12-hour troponin he was discharged.

A few hours later he re-attended A&E with urinary retention and persistence of the ‘wobbly’ legs sensation. Neurological examination revealed proximal lower limb weakness, with MRC grade 4- at both hip flexors, but MRC grade 5 in all other lower limb muscle groups. Pin prick was normal in all dermatomes. This injury was classified as AIS D.

Magnetic resonance imaging revealed a linear area of high signal within the anterior aspect of the cord from T4 to T7. Other investigations including thrombophilia and vasculitis screens were normal. A diagnosis of SCI was made and he was commenced on aspirin and LMWH. Eight months later he had regained full muscle strength, but had persisting bladder dysfunction requiring intermittent self-catheterisation.

Seven years following presentation he only requires intermittent catheterisation on an ad hoc basis. He required medical management for erectile dysfunction. He returned to full time work.

### Case 3

A 64-year-old male, with a longstanding history of hypertension, intermittent claudication and smoking 40 cigarettes per day, developed altered sensation from his abdomen to his feet and severe leg weakness. Examination showed a flaccid paraparesis, with MRC grade 0 in all lower limb muscle groups, intact proprioception and a sensory level at T6. Due to the absence of sensory and motor function in S4-S5 this was classified as AIS A.

MRI showed signal change in the lower spinal cord, most marked at the conus extending superiorly to T9. He was commenced on LMWH but eight months later he remained wheelchair-bound. He required assistance of one person for all functional transfers, and intermittent self-catheterisation and alternate day bowel care with an enema and digital rectal evacuation.

Four years after presentation he had his last review at the SCIU. He remained wheelchair bound. He had required prolonged periods of bed rest, due to pressure ulcers and osteomyelitis of the left ischium, and an indwelling catheter. Five years after presentation this gentleman passed away during an inpatient admission with sepsis.

### Case 4

A 53-year-old female with a history of essential hypertension developed mild lower back pain which progressed within minutes to a more severe pain, with weakness and numbness of her legs which she described as an ‘epidural sensation’. She presented to A&E in urinary retention and examination revealed a partial Brown-Séquard syndrome with a sensory level at L3 on the right side. In her right lower limb she had MRC grade 3 power in hip flexion and grade 2 in all other muscle groups, and in her left limb she had MRC grade 2 power in hip flexion and grade 0 in all other muscle groups. This injury was classified as AIS C.

She was imaged on the day her symptoms began but no abnormality was detected. Her clinical findings remained unchanged and on day six repeat imaging revealed an abnormality at the conus. She quickly recovered strength in her right leg, and by day four had grade 5 power. Her left leg was grade 1 in all but knee extension, which was grade 4. By the time of discharge from SCIU, she was mobile with a rollator and could ambulate up to 100 m. She continued to require intermittent self-catheterisation and digital rectal evacuation.

Ten years following presentation she was still under review with the SCIU team. She was now mobile with a single axis cane. Manual rectal evacuation was still required. There were problems with urinary incontinence, and recurrent UTIs, despite surgery for vaginal prolapse and stress incontinence.

### Case 5

A 56-year-old renal transplant patient developed acute back pain followed within five hours by urinary retention and difficulty standing. The next morning he was unable to walk. Examination revealed a complete paraplegia (MRC grade 0 in all lower limb muscle groups), flaccid tone and loss of reflexes. He had complete loss of pain and temperature sensation at T7 and below. With a complete absence of sensory and motor function in S4-S5 this was classified as AIS A.

He had developed renal failure 29 years previously, the aetiology of which was unclear, and received haemodialysis until a successful renal transplant in 1996. His blood pressure had been well controlled at recent clinic visits. His MRI revealed abnormal high signal in the classical watershed area of the spinal cord from T4 to T6. Two months later, on discharge from SCIU, his paraplegia remained complete.

On final review with the SCIU team, four years following presentation, his status remained essentially unchanged, with the exception of the requirement of an indwelling urethral catheter. Three years following this review, he passed away during a hospital admission with bronchopneumonia.

## Discussion

The spinal cord receives blood supply from a single anterior spinal artery (ASA) and two smaller, paired posterior spinal arteries [14]. The ASA arises superiorly from the union of the vertebral arteries at the level of the foramen magnum and supplies the anterior third of the spinal cord [14, 15]. The blood supply of the lower thoracic and lumbar regions of the cord is reinforced by a large, single anterior radicular artery—the artery of Adamkiewicz [15]. The cervical and upper thoracic portions are most vulnerable to ischaemia due to their lack of an equivalent arterial supply and, therefore, may be referred to as watershed regions [6, 16]. This correlates with our findings, as MRI in cases 1, 2 and 5 demonstrated ischaemic changes in the spinal cord region between C5 and T8.

Occlusion of the ASA by an atherosclerotic plaque or thrombus can lead to anterior SCI. The relationship between vascular risk factors and SCI has previously been documented [5, 9, 10, 12, 17, 18]. We hypothesise that the aetiology in cases 3, 4 and 5 is arteriosclerosis associated with vascular risk factors. Cases 3 and 4 were both over the age of 50 years and had a history of hypertension and smoking, while case 5 had a long history of chronic kidney disease as the likely predisposing factor for accelerated atherosclerosis. Although thrombophilia can lead to SCI, we do not believe it was the cause in our cases. Case 1 may have been at risk of venous infarction from a pregnancy-related hypercoagulable state, but her deficit was in the classical ASA distribution—suggesting arterial pathology.

The aetiology in case 2, who had probable prodromal symptoms and made the best recovery, remains elusive. A dural arteriovenous fistula is a possibility. These malformations are more common in males [19], and patients typically have pain which can mimic sciatica and may worsen after exercise [20].

Patients in whom the aetiology remains obscure are not unusual. Weidauer et al. [4] described 55 patients with SCI and considered that in 23.5% of these the aetiology was unclear. Castelló et al. [5] were unable to establish a cause in 29.3% of 42 SCI cases, and a recent review of 526 cases from 14 different studies by Batsou et al. [17] found that in 24–74% of patients the aetiology remains unclear. Older studies reported ever higher rates of unknown aetiology [21–23], perhaps due to less advanced diagnostic techniques.

The outcome of SCI is variable and usually depends on numerous factors, but most patients have a poor functional recovery and reduced life expectancy. The studies by Ge et al. [11] and Castelló et al. [5] of 30 and 41 cases, respectively, both found that a more severe initial presentation (for example, paraparesis) was associated with poorer long-term outcomes. Stenimahitis et al. [12] analysed 30 cases of SCI (median follow-up 2.1 years) and concluded that severity of initial presentation was an independent outcome predictor. Our findings are consistent with these data, as cases who scored lower on the AIS – indicating a larger degree of neurological impairment at presentation, received a higher rating on the modified Rankin Scale (mRS) at long-term follow-up – indicating greater disability (Table 1).

In addition to injury severity at presentation, older age, vascular risk factors and involvement of ASA watershed areas are negative predictors for functional outcome in the setting of SCI [5, 6, 24]. This is supported in our findings, as cases 3, 4 and 5, who were all over 50 years of age with risk factors for arterial disease, demonstrated poorer functional status at last follow-up. Factors affecting rehabilitation in SCI are not well documented in the literature, but when considering spinal rehabilitation it has been shown that a greater delay between symptom onset and starting rehabilitation is associated with poorer outcomes [25].

In a study by Ge et al. [11] the long-term follow-up (mean 2.7 years) of 30 patients with SCI found that 57% were still wheelchair bound, and 23% were ambulating with assistance. A more recent study by Pikija et al. [18] of 88 cases of SCI reported that 36% required a wheelchair for ambulation and 16% required walking aids.

Despite being a relatively small case series, our findings on long-term functional outcome correspond with these data. Our last follow-up (mean 6.5 years) of five patients demonstrated 40% were wheelchair-bound and 20% required ambulatory assistance. Additionally, 60% required catheterisation (Table 2). One of our patients (case 1) improved significantly, becoming independently mobile despite a severe initial presentation.

All our cases presented with some combination of acute back or chest pain, motor, sensory and autonomic dysfunction. Case 2 initially experienced symptoms akin to acute coronary syndrome with a potential prodrome period in the months prior to presentation. His long-term functional outcomes were noticeably superior to those of cases 3, 4 and 5 (Table 1). It is possible that these differences are linked to his younger age at presentation and lack of vascular risk factors. The importance of correcting vascular risk factors in the management and prevention of SCI has previously been documented [12, 17]. This highlights that although SCI can be attributed to arterial pathology, this condition is multifactorial and treatment and rehabilitation require an individualised approach [12].

Our case series following patients with SCI illustrates that although non-traumatic SCI is less common, the risk of spinal cord ischaemia secondary to vascular disease is not negligible. Additionally, our cases demonstrate that a more severe neurological presentation is associated with poorer long-term functional outcomes, despite extensive spinal rehabilitation. Early diagnosis appears essential to exclude acute cord compression and improve long-term outcomes.

## Data Availability

All data supporting the findings of this report are available within the article and its supplementary files.
